# Leiomyosarcoma of Bone: A Case Report

**DOI:** 10.1155/2011/980257

**Published:** 2011-11-17

**Authors:** Andreas Frings, Andreas Leithner, Bernadette Liegl-Atzwanger

**Affiliations:** ^1^Department of Orthopaedics and Orthopaedic Surgery, Medical University of Graz, Auenbruggerplatz 5, 8036 Graz, Austria; ^2^Institute of Pathology, Medical University of Graz, Auenbruggerplatz 25, 8036 Graz, Austria

## Abstract

The aim of this paper is to present clinicopathologic features and immunohistochemical findings of a primary leiomyosarcoma of bone occurring in the proximal femur of a 46-year-old Caucasian male patient. Each case report on this exceedingly rare entity contributes to the notion of this disease.

## 1. Introduction

Sarcomas comprise a heterogeneous group of mesenchymal neoplasm. The vast majority of so-called smooth-muscle tumors arise in the uterus, gastrointestinal tract, mesentery, and omentum [1]. Leiomyosarcomas are malignant smooth-muscle tumors composed of spindle cells showing distinct smooth muscle features that exceedingly rarely occur in the bone [2, 3]. Despite of an unknown aetiology, they typically occur in middle-aged or older persons [1]. Leiomyosarcoma of the bone is reported to occur predominately in male adults, particularly in large bones, most commonly in the distal femur [4]. First reported by Evans and Sanarkin in 1965 [3], this entity is thought to arise from the vascular smooth-muscle cells within the bone [4].

For nonretroperitoneal soft tissue leiomyosarcomas, local recurrences and metastases usually occur within the first few years after initial diagnosis and commonly metastasize to the lungs. Prognosis depends on the location, stage, and grade of the primary tumor as well as the presence of metastatic disease [1].

Herein we present clinicopathologic features and immunohistochemical findings of a primary leiomyosarcoma of bone occurring in the proximal femur of a 46-year-old Caucasian male patient.

## 2. Case Presentation

A 46-year-old Caucasian man was admitted to our department due to pain in his right proximal thigh. Medical history revealed no contributory family and past histories, and no neurological deficits were detected by physical examination.

**Figure 1:**
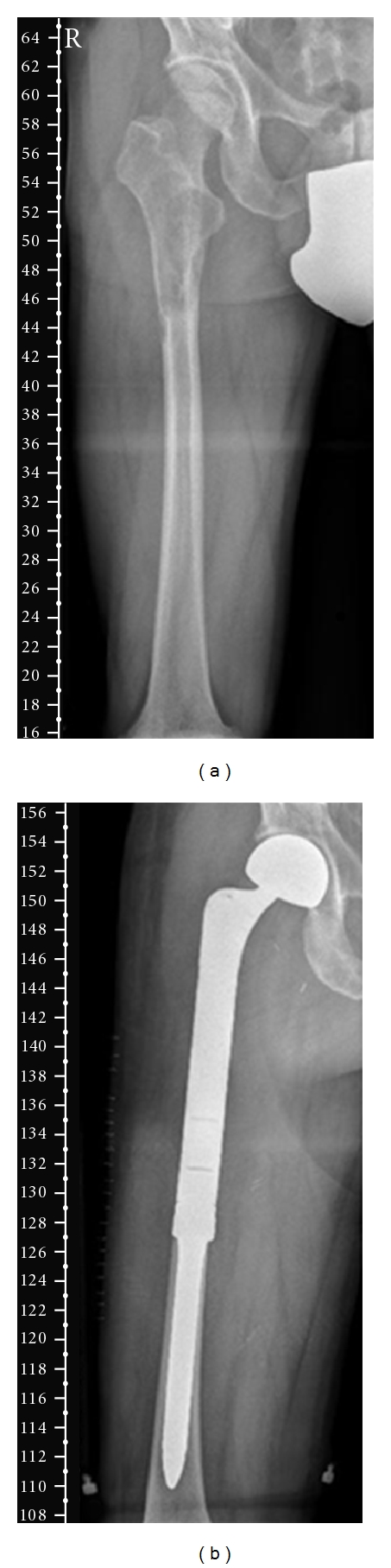
Radiographs of the patient's right femur: (a) preoperative, an expanding lytic lesion (10 cm in max. diameter) located in the right proximal femur with extension into the proximal shaft, (b) two months after limb salvage surgery followed by endoprosthetic replacement.

X-rays and MRI presented an expanding 10 cm lytic lesion with an inhomogeneous contrast medium enhancement occupying the right proximal femur and extended into the proximal shaft (Figure 1). The lesion showed amorphous density resulting from a high-grade destruction of the cancellous bone. A perforation of the osseous cortex with an adjacent extraosseous mass was detected. Subsequently performed tumor staging including soft tissue scans of chest, abdomen, and pelvis gave no evidence of metastatic disease or evidence of another primary tumor.

Biopsy specimen revealed a malignant mesenchymal fascicular spindle cell proliferation with smooth-muscle differentiation reflected by the expression of a-SMA (a-smooth-muscle-actin), desmin, and caldesmon by immunohistochemistry. The patient underwent limb salvage surgery followed by endoprosthetic replacement (Figure 1). Wide surgical margins were achieved and after a 23-day inpatient stay, the patient was discharged from hospital with postoperative chemotherapy according to the EUROBOSS study protocol. 

**Figure 2:**
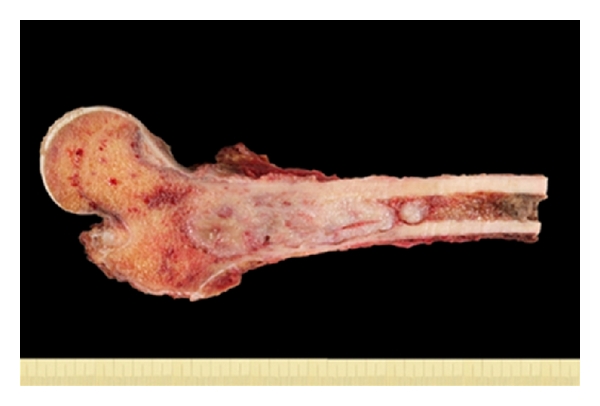
The explant shows a proximal femur measured 25 cm in length, a tumor of 10 cm in max. dimension and predominantly grey-white appearance occupies the medullary cavity.

The explant consisted of a 25 cm long proximal femur (Figure 2) with a 10 cm tumor in largest diameter that mainly occupied the medullary cavity. An area of cortical perforation with an extraosseous soft tissue mass of 2 cm was seen. The tumor showed a grey-white cutting surface (Figure 2). 

**Figure 3:**
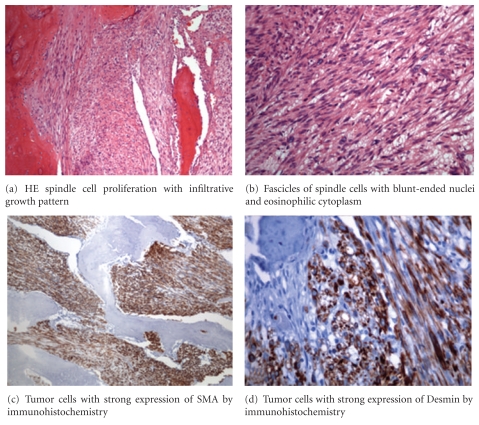
Histologic workup of the tumor tissue: (a) HE, (b) HE, (c) SMA, (d) Desmin.

 The histological evaluation revealed a malignant fascicular spindle cell proliferation with an infiltrative growth pattern composed of interweaving fascicles. The tumor cells were spindle shaped, elongated, blunt ended, hyperchromatic, focally pleomorphic, and surrounded by a prominent eosinophilic cytoplasm (Figure 3). With antibodies against a-SMA, desmin, and caldesmon the majority of the tumor cells showed a specific positive reaction. Immunohistochemistry using antibodies directed against Cytokeratin and S-100 were negative (Figure 3). In the area of the previous biopsy site a spot of 0.2 mm of cartilaginous differentiation was found.

 After one year of followup, a successfully performed one-stage revision surgery became indispensable due to a late-onset infection of the primary prosthesis. For more than 9 months of followup after first surgery no evidence of metastatic disease or local recurrence was seen. Although two 2 mm lesions were detected in both lungs by soft tissue scans the lesions did not show any progression during the following 16 month of followup.

## 3. Discussion

Primary leiomyosarcoma of bone is exceedingly rare, accounting for less than 0.7% of all primary malignant bone tumors [5]. It most commonly occurs in male adults, particularly in large bones like the distal femur [3, 6, 7]. Although prognosis has been difficult to define due to its rarity, most studies have indicated that these tumors are of an aggressive nature [8].

As primary leiomyosarcoma of bone is exceptionally rare we considered the following differential diagnoses. First of all skeletal metastases from a primary leiomyosarcoma located in the gastrointestinal tract or soft tissues were excluded by CT scans and scintigraphy using the diagnostic guidelines described by Antonescu et al. [9].

In addition low-grade intramedullary osteosarcoma, fibrosarcoma of bone, and metastases of a spindle cell carcinoma were excluded by means of morphological analysis and immunohistochemistry.

Based on a 0.2 mm area of cartilaginous differentiation a dedifferentiated chondrosarcoma (DCS) was considered as another differential diagnosis. DCS is defined by the presence of a well-differentiated cartilaginous component with abrupt transition in a dedifferentiated tumor component, which may have features of pleomorphic sarcoma, NOS (malignant fibrous histiocytoma, osteosarcoma, fibrosarcoma), and exceptional rarely leiomyosarcoma [10]. Typically DCS is seen in older individuals than in the patient presented herein, with an average age between 50 and 60 years [10]. DCS frequently has a typical radiological appearance with a characteristic bimorphic radiological pattern reflecting the two differentiation levels of the tumor [11]. Herein, imaging examination revealed a malignant appearing, permeative lytic lesion located near the metaphysic portion of the bone. No radiological signs of a cartilaginous component in terms of a bimorphic pattern or stippled calcifications were seen as they would have been expected in a DCS.

Approximately 90% of all patients with DCS have a poor prognosis and die thus due to metastatic disease within the first two years after diagnosis [10, 12–14]. The small lesions detected in the lungs of our patient were not growing over a period of 16 months—suggesting that metastatic disease is unlikely in our case.

The age of our patient, the radiologic appearance (lack of a bimorphic pattern, lack of punctate or ring-like opacities) as well as the fact that the 0.2 mm area with cartilaginous differentiation corresponded to the area of the previous biopsy side prompted us to consider the cartilaginous component as reactive, and therefore, the differential diagnosis DCS was rejected.

We herein report clinicopathologic features and immunohistochemical findings of a primary leiomyosarcoma of bone located in the proximal femur of a 46-year-old Caucasian male patient. Each case report on this exceedingly rare entity contributes to the notion of this disease. As the reported case shows, clinicians should be aware of leiomyosarcoma of bone as differential diagnosis.

## References

[1] Somerhausen NDSA, Cin PD, Fletcher CDM, Unni KK, Mertens F (2002). Smooth muscle tumours. *Pathology and Genetics of Tumours of Soft Tissue and Bone. World Health Organization Classification of Tumours*.

[2] Petra M, Gibbons CLMH, Athanasou NA (2002). Leiomyosarcoma of bone arising in association with a bone infarct. *Sarcoma*.

[3] Evans D, Sanarkin NG (1965). Primary leiomyosarcoma of bone. *Journal of Pathology & Bacteriology*.

[4] Jundt G, Moll C, Nidecker A, Schilt R, Remagen W (1994). Primary leiomyosarcoma of bone: report of eight cases. *Human Pathology*.

[5] Johnson S, Tetu B, Ayala AG, Chawla SP (1986). Chondrosarcoma with additional mesenchymal component (dedifferentiated chondrosarcoma): I. A clinicopathologic study of 26 cases. *Cancer*.

[6] von Hochstetter AR, Eberle H, Rüttner JR (1984). Primary leiomyosarcoma of extragnathic bones. Case report and review of literature. *Cancer*.

[7] Myers JL, Arocho J, Bernreuter W, Dunham W, Mazur MT (1991). Leiomyosarcoma of bone. A clinicopathologic, immunohistochemical, and ultrastructural study of five cases. *Cancer*.

[8] Khor TS, Sinniah R (2010). Leiomyosarcoma of the bone: a case report of a rare tumour and problems involved in diagnosis. *Pathology*.

[9] Antonescu CR, Erlandson RA, Huvos AG (1997). Primary leiomyosarcoma of bone: a clinicopathologic, immunohistochemical, and ultrastructural study of 22 patients and a literature review. *American Journal of Surgical Pathology*.

[10] Milchgrub N, Hogendoorn PCW, Fletcher CDM, Unni KK, Mertens F (2002). Cartilage tumours. *Pathology and Genetics of Tumours of Soft Tissue and Bone. World Health Organization Classification of Tumours*.

[11] Littrell LA, Wenger DE, Wold LE (2004). Radiographic, CT, and MR imaging features of dedifferentiated chondrosarcomas: a retrospective review of 174 de novo cases. *Radiographics*.

[12] Capanna R, Bertoni F, Bettelli G (1988). Dedifferentiated chondrosarcoma. *Journal of Bone and Joint Surgery—Series A*.

[13] Dahlin DC, Beabout JW (1971). Dedifferentiation of low-grade chondrosarcomas. *Cancer*.

[14] Mitchell AD, Ayoub K, Mangham DC, Grimer RJ, Carter SR, Tillman RM (2000). Experience in the treatment of dedifferentiated chondrosarcoma. *Journal of Bone and Joint Surgery—Series B*.

